# Life satisfaction favors reproduction. The universal positive effect of life satisfaction on childbearing in contemporary low fertility countries

**DOI:** 10.1371/journal.pone.0206202

**Published:** 2018-12-05

**Authors:** Letizia Mencarini, Daniele Vignoli, Tugba Zeydanli, Jungho Kim

**Affiliations:** 1 Dondena Centre for Research on Social Dynamics and Public Policy, Department of Management and Technology, Bocconi University, Milan, Italy; 2 Department of Statistics, Computer Science, Applications, University of Florence, Florence, Italy; 3 Department of Economics, Martin Luther University of Halle-Wittenberg, Halle (Saale), Germany; 4 Department of Economics, Ajou University, Suwon, South Korea; 5 IZA, Bonn, Germany; Kyoto University, JAPAN

## Abstract

Do people with higher life satisfaction have more children? Having children requires considerable energy and investment on the part of parents. However, even in countries where contraceptives are easily available and widely used, where having children is optional and most of time the result of an intended action, parenthood has not gone “out of fashion”. This paper tests the hypothesis that higher life satisfaction fosters reproductive behavior. We argue that people satisfied with their overall life feel better prepared to start the monumental task of childrearing. If, it is suggested, life satisfaction facilitates fertility, then this positive link should be observable in contemporary low fertility societies. The hypothesis is tested by taking overall life satisfaction as a determinant of fertility behavior using long longitudinal data available for developed countries: namely for Australia, Germany, Russia, South Korea, Switzerland, the United Kingdom, and the United States. We find that higher levels of subjective well-being are, indeed, associated with a higher probability of having children in all the countries considered. We, therefore, conclude that life satisfaction favors reproduction, at least in low fertility societies.

## Introduction

Can life satisfaction influence the decision to have children? In their review of the literature, which begins with the 1970s, Kahneman, Diener, and Schwarz [1] acknowledged that a causal link between subjective well-being and demographic behavior might be intuitively appealing, but that it was by no means certain. They concluded that the demographic consequences of subjective well-being should be a priority in research.

In contemporary developed societies, with a high prevalence of contraceptives and low fertility, childbearing is generally an outcome of couples’ decision making. Under the realistic assumption that having a child is the result of a reasoned choice [2, 3], the question of whether reproductive choices represent a direct function of subjective well-being becomes a natural one. However, the question has not been systematically dealt with in the literature [4]. Indeed, this question has received far less scholarly attention than the reverse question, namely, the possible effects of fertility on subjective well-being (for a review see [5]).

There is a keen interest in how subjective well-being (intended here as a broad category, covering both affective aspects, i.e. positive and negative feelings and expressions of happiness, as well as cognitive assessments about life satisfaction [6, 7]), connects to fertility behavior–i.e. the actual reproductive performance. From a purely theoretical perspective, subjective well-being can have a neutral or a negative or a positive link with fertility [8].

According to the “affective forecasting theory” people base their decisions on affective forecasts, i.e. their predictions about their own emotional reactions to future events [9, 10]. When people decide to have a child they anticipate a happy event and, therefore, positive consequences for their satisfaction with life. Both folk theories about childbearing [11] and a rise in subjective well-being before childbirth, typically found in life satisfaction or happiness trajectories before and after the birth of a child, confirm a positive affective forecast (e.g., [12]). In this sense the level of subjective well-being before the conception of a child should not matter much, since at any level of personal satisfaction the future positive event of a birth would be seen as a way to enhance one’s well-being. Even those who have a low level of subjective well-being could decide to have a child as a (predicted) means to improve their well-being. In the same way, unhappy couples might opt for childbearing as a means to improve their relationship [13, 14]. Consistently, but for quite contrary reasons, a negative relationship between subjective well-being and fertility is suggested by an aversion to lifestyle change, something mentioned by many voluntarily childless persons [15, 16]. In other words, for some, children may threaten life satisfaction. Consequently, for these individuals, high levels of subjective well-being tend to mean lower fertility due to competing priorities [17]. However, even if this kind of motivation is proven and significant, it is likely to be confined to a small proportion of the population, as childless people at the end of their reproductive cycle rarely account for more than ten to fifteen percent of the total.

Instead, there are compelling motivations linking, first, lower subjective well-being to lower fertility and, second, higher subjective well-being to a higher likelihood of having a(nother) child. Low levels of subjective well-being are often characterized by depression and stress, symptoms linked to negative affective forecasting [18], i.e. a more pessimistic view of the future. Unsurprisingly, then, several studies have found that low subjective well-being, measured specifically in terms of depression and stress tends to lower fertility. The effect is significantly linked to reduced fecundity, and higher numbers of miscarriages and stillbirths (i.e., [19, 20]).

On the other hand, in low fertility countries, childbearing is now very much viewed as a choice that individuals make as part of a process of self-realization [21, 22]. A key assumption in much of the fertility literature (though this is not always expressed explicitly) is that individuals’ decision-making depends on their quest for higher well-being–in accordance with the prevalence of positive affective forecasting. Childbearing, especially when it is an outcome of extensive planning [23], competes with many other life goals, which also matter for life satisfaction. Hobcraft and Kiernan [24] argue that there are five “basic requirements” for deciding to have a child. The potential parents: will want a partner; want to have completed their education; be employed; have satisfactory housing; and enjoy a “sense of security”. Drawing on that argument, Billari [4] extended the notion of “sense of security” to subjective well-being, conceptualizing it in terms of psychological security. He suggests that both happiness (i.e. a measure of affective subjective well-being) and life satisfaction (i.e. the cognitive part of subjective well-being) naturally predispose an individual to childbearing. These arguments suggest that the likelihood of childbearing is a direct function of individuals’ subjective well-being. Intuitively it makes sense that satisfied people feel better prepared to start the monumental task of childrearing. In addition, fertility may come about because having a partner contributes to a person’s satisfaction with life, which would naturally affect fertility positively, at least if the relationship is a good one [25].

The positive effect of subjective well-being on fertility behavior can be traced in the few studies that have included subjective well-being as a determinant of fertility. The findings, which have been quite consistent, seem to support the idea that, both for women and men, individual subjective well-being–sometimes measured as happiness, sometimes as overall life satisfaction–represents an important goal to reach before making the decision to have children. In this vein, Billari [4] indicated that happier people are more likely to *intend* to have a(nother) child. Perelli-Harris [26] showed that in Russia, subjective well-being is positively linked to wanting and having additional children. Parr [8] found that life satisfaction is a determinant of fertility in Australia and, for both sexes, there is a strong positive relationship between prior satisfaction with life and fertility two years later. Le Moglie, Mencarini, and Rapallini [27] suggested that an increase in life satisfaction might, indeed, result in the increased likelihood of having a second child in Germany. Cetre et al. [28] found that a positive association between childbearing and subjective well-being only exists in developed countries where fertility is largely optional. Then–using the longitudinal German data–they provided evidence for a positive selection into parenthood, whereby happier people are more likely to have children.

Our expectation is that the positive effect of subjective well-being on fertility would, therefore, dominate in the context of developed countries, where the level of fertility is relatively low, as are unplanned births. To test this hypothesis we make here a systematic analysis of available longitudinal surveys across developed countries to establish whether higher subjective well-being is, indeed, related to the probability of having a(nother) child in low fertility societies. We extend previous research in two critical ways. First, the few studies that explore the effects of subjective well-being on fertility are country specific. Instead, we systematically inspect the link between life satisfaction and fertility across seven rich societies. All seven have below replacement fertility levels, with the average number of children *per* woman standing between 1.5 and 1.8 (Population Bureau Reference in 2016): Australia, Germany, Russia, South Korea, Switzerland, the United Kingdom, and the United States. We hypothesize that, if life satisfaction facilitates fertility, this positive link should be observable in any contemporary low fertility society.

Second, prior research has largely overlooked how the relationships between subjective well-being and fertility is likely to differ between first and subsequent births. Clearly, for those who have already had children, the expected happiness or life satisfaction from having additional children may be affected by their own and their partner’s childbearing and childrearing experiences and, particularly, post-birth subjective well-being. Hence, only the effect of subjective well-being on the likelihood of having the *first* child embodies the “net effect of subjective well-being on fertility”. After the birth of the first child, however, the level of parental subjective well-being also incorporates parental subjective well-being associated with previous child(ren), which critically depends on their objective and perceived costs. Most economic studies fail to conceptualize and operationalize fertility choices as a succession of parity transitions over one’s life course [29], typically summarizing fertility as a single outcome variable, such as the total number of children born (e.g., [30–34]). We thus extend previous research by systematically scrutinizing the effects of the cognitive aspect of subjective well-being, i.e. overall life satisfaction, on the probability of having a child in general, as well as on the probability of having a first and a second child.

Our study offers new empirical evidence for the impact of subjective well-being on fertility. We incorporate subjective well-being as a determinant of fertility behavior, and present a systematic and robust analysis based on the established and long-running longitudinal datasets currently available for developed countries.

## Materials and methods

### Data

We used available long longitudinal datasets for the developed world. These came from seven countries, namely Australia, Germany, Russia, South Korea, Switzerland, the United Kingdom, and the United States.

All of these surveys are longitudinal panels (i.e. the same individuals are interviewed in every round), nationally representative, and provide individual-level and household-level information on a wide range of issues. For Australia we use HILDA (Household, Income and Labour Dynamics in Australia Survey), an on-going longitudinal survey which has been conducted yearly since 2001, and in which all adult members are interviewed in each household. Here we use the first twelve waves until 2012. HILDA provides information about economic and subjective well-being, labor-market and family dynamics. For Germany, we use the SOEP (Socio-Economic Panel) from its start in 1984 up to 2012 (though note that other waves have been collected). The SOEP offers a representative sample of the entire German population, with information on individual life histories, such as career path, marital status, childhood biography, social background, and immigration history. For Russia we use RLMS (Russian Longitudinal Monitoring Survey) from 1995 to 2014. The panel is conducted yearly and takes in about 10,000 individuals in 4,000 households and gathers information on a set of social and demographic data at the family and individual level. It is the first national probability sample carried out in the Russian Federation. For South Korea we use data from five waves of KLIPS (Korean Labor and Income Panel Study) from 2009 to 2015. It is an ongoing longitudinal survey with a representative sample of Korean households. It has, since 1998, annually tracked the demographic and economic characteristics of these households, as well as the economic activities, labor movement, income, expenditure, education, job training, and social activities of individuals in said households. The original sample consisted of 5,000 urban households, but it was expanded to 6,721 households in 2009 covering the entire Korean population. For Switzerland, we use thirteen waves, from 2000 to 2012, of the SHP (Swiss Household Panel), a yearly panel study on the dynamics of living conditions at both individual and household-level. It offers information for broad categories such as the labor market, employment, income, poverty, living conditions, quality of life, health, and physical activity. For the United Kingdom we use the BHPS (British Household Panel Survey) from 1996 to 2008. The BHPS provides information on individual, household, and job-employer related subjects. Initially 5,000 households were interviewed in the UK. Additional samples of 1,500 households in Scotland and Wales were added to the main sample in 1999, and, in 2001, 2,000 households were added in Northern Ireland, making the panel suitable for UK-wide research. The BHPS ended in 2008, and, in 2009, a new British panel survey called “Understanding Society” started. Only a small portion of BHPS individuals continued to be interviewed in the new survey. To ensure a long time span, we use here the original BHPS. Finally, for the United States we use two waves of the biennial panel of PSID (Panel Study of Income Dynamics), one from 2009 and the other from 2011. The survey has been collecting, since 1968, economic, social, demographic, health, geospatial, and psychological data. But information on life satisfaction was only added in 2009.

Other panel surveys exist for other developed countries, but those chosen here include repeated measures of subjective well-being and birth histories for national representative samples.

Our study includes only men and women in their reproductive age. Consequently, the respondent must be older than 20 and younger than 50, and have reported valid answers for the variables included in the analysis. After imposing the age restriction, and deleting missing information, we ended up with the following sub-samples: for Australia 88,181 (out of 165,298) person-year observations (i.e. the product of the number of years times the number of individuals “at risk”, which means, in this case, in reproductive age); 258,779 (out of 693,055) for Germany; 108,895 (out of 249,793) for Russia; 31,971 (out of 70,392) for South Korea; 38,561 (out of 143,239) for Switzerland; 80,762 (out of 603,558) for the United Kingdom; and, 20,743 (out of 802,685) for the United States.

### Statistical model

The relationship between subjective well-being and childbearing can be discussed in a dynamic perspective, where the level of subjective well-being in a period may affect the likelihood of having a child in the next period. A general approach would be to assume that a sexually-active woman (or a couple), by not using contraception, actively seek to have a child, where the actual birth is denoted by *b*_*t*_. Since the outcome is dichotomous, a natural choice would be to employ a Probit model for the empirical analysis. The probability of having a child might be described as a function of life satisfaction, *LS*_*t-1*_ and a range other variables denotes as *z*_*t-1*_ Mathematically:

$$Prob\;\left[b^{*}{}_{t}=1|LS_{t‑1,}z_{t‑1}\right]=\Phi \;(\beta LS_{t‑1}+\gamma ’\;z_{t‑1})$$

where Φ indicates the cumulative distribution function of the normal distribution.

We ran regressions separately for men and women, as we expected the relationship between childbearing and subjective well-being to be gender specific in several dimensions. The effects of the explanatory variables for the decision to both marry and having children may be different for men and women. They might be different both because of biological differences (i.e. age for example) but also because of the gender roles one may find across and within societies. That is not just for subjective well-being; other observable variables will potentially have different effects on the chance of having a child. For instance, income might matter more for men than women, in terms of deciding to have a child. Consequently, it would be appropriate to estimate the statistical model separately for men and women—an approach that is also consistent with the demography literature.

We proposed four model specifications. Model 1 is a regression where all country samples are pooled together, but where estimation is done separately for not only men and women, but also for the probability of having a child, the probability of having a first child, and finally the probability of having a second child. The key variable is the measure of life satisfaction as described in the next section. Next the model includes dummy variables for each of the countries (i.e. country-fixed effects) in order to control for country specific differences in the probability of having a(nother) child, and interaction terms between the country dummies and the measure of life satisfaction. The interactions measure the extent to which life satisfaction potentially affects fertility differently across countries. In Models 2, 3 and 4 the regressions are repeated, but they are now done separately for each country. We clustered standard errors at the household-level, which means that we account for serial correlations in the error structure.

For our analysis we use the software STATA version 14 and apply the probit statistical procedure for all regressions.

### Variables

As a dependent variable, we utilized a dummy variable of the child-birth event, indicating whether or not there had been any births between waves. For each individual present in two consecutive waves, we observed his/her characteristics before the birth of the child. Due to the natural length of pregnancy, we include subjective well-being in our model and all the individual characteristics lagged by at least one year with respect to the birth. More specifically, since the interview date changes at the individual level in every year, we lag values accordingly, i.e. for each individual, we calculate nine months back from the birth date of the child in survey round at time t and, if this falls before that of the interview at time t-1, we further lag all the variables back to time t-2. In the cases where the month of the birth of the child is not available, we always lagged the variables back to time t-2. An important feature of this construction is that it eliminates the possibility of capturing the anticipation effect driven by the change in life satisfaction due to a pregnancy [27]. In fact, even if the anticipation effect might be present among individuals who, at some point in their life have decided to have a child, it is reasonable to assume that the effect only materializes at the moment in which the pregnancy is discovered.

Our key explanatory variable was derived from the life satisfaction question, which is an indicator of individuals’ cognitive subjective well-being. The wording of the questions for overall individual life satisfaction is similar and comparable in the seven surveys, whereas the scale of answers varied. We, therefore, adjusted subjective well-being on a 0–10 scale to achieve consistency across subjective well-being measures. The life satisfaction in the BHPS data set is based on the question “How dissatisfied or satisfied are you with your life overall?”. This allowed a seven-point scale ranging from 1 (not satisfied at all) to 7 (completely satisfied). This variable is available from wave-6 onwards, except wave-11, so we can utilize 12 waves. The life satisfaction question in the SOEP asks: “How satisfied are you with your life, all things considered?”, with responses given on a 0–10 scale, in which 0 is “completely dissatisfied” and 10 is “completely satisfied”. Similarly, in the Swiss survey the question is: “In general, how satisfied are you with your life, if 0 means “not at all satisfied” and 10 means “completely satisfied”?”. In the HILDA survey, the life satisfaction question is designed as follows: “In general, how satisfied are you with your life if 0 means ‘not at all satisfied’ and 10 means ‘completely satisfied’?”. We start with wave 2 because the life satisfaction question was not available in the first wave. In PSID, the life satisfaction question is: “Please think about your life as a whole. How satisfied are you with it?”. The accompanying scale is from 1 to 5, with 5 being the least satisfied. In the KLIPS, the life satisfaction question is: “Overall, how satisfied or dissatisfied are you with your life?” Individuals are asked to respond on a scale from 1 (“very satisfied”) to 5 (“very dissatisfied”). In the RLMS we extracted the individual subjective well-being score from the life satisfaction question: “To what extent are you satisfied with your life in general at the present time?”: 1 stands for “fully satisfied” and 5 stands for “not satisfied at all”. For reasons of comparison, we reversed the order of the last three surveys listed here (i.e. PSID, KLIPS and the RLMS).

For the analysis of the association between life satisfaction and subsequent fertility, it is necessary to control for a range of other variables, which may affect both life satisfaction and fertility. As for the basic demographic characteristics, the effect of age needs to be controlled for, in view of the documented variation in life satisfaction over life course, and the variation in fertility with age [35]. We group individuals’ age in three categories (0 = 20–29; 1 = 30–39; 2 = 40–49 years old). Since living in a co-resident union may affect both fertility and life satisfaction, we included partnership status in the equation [36–39]. The union status is a dummy variable that takes value 1 if the individual is in a co-resident union (irrespective of whether he/she is married or cohabiting), otherwise 0. In the general model predicting the probability of having a child, we additionally accounted for the number of children (0 = no child; 1 = one child; 2 = two or more children). We also included the age of the youngest child (continuous variable up to age fifteen) in the model predicting the probability of second children.

Clearly, socio-economic factors also needed to be controlled for, since income, having or not having a job, and education level have also been shown to affect both life satisfaction and fertility [8, 36, 40–41]. We collapsed the levels of education into three broad groups: primary education (0), secondary education (1), and tertiary education (2). As for employment status, we considered a dummy variable indicating whether the individual is employed (1 = employed; 0 = otherwise). The calculation of the income-quintile variable was based on equivalized income (i.e. total household income is divided by the sum of the weightings to yield a representative income) with the OECD modified equivalence scale (i.e. assigning 1 to the first adult, 0.5 to the second and each subsequent person aged 14 and over, and 0.3 to each child under fourteen). When individuals are in a couple, we also controlled for the partner’s level of education and employment status (categorized in the same way as the respondent’s education and employment status).

To acknowledge within-country differences, all specifications included regional dummies. For Germany, Switzerland, and the United Kingdom we considered the level 1 of the NUTS classification (Nomenclature of Territorial Units for Statistics); for Russia we utilized the federal districts; for Australia we considered the 2001 ASGC (Australian Standard Geographical Classification) units; for the United States we relied on the Census Bureau-designated regions and divisions; for South Korea, meanwhile, we used a division of provinces and cities, totaling sixteen geographical units. Finally, all specifications included year-of-survey dummies.

To verify whether the variables we selected are collinear to one another, we produced: 1) a correlation matrix for each dataset (results available upon request from authors). The correlations among variables are quite low, ensuring that the properties of the estimator remain unaltered; 2) a Variance Inflation Factor analysis (VIF) for all the independent variables (results available upon request from the authors). Consistently with the correlation matrix, the average VIF value is 1.5 (and never above 5).

## Results

**Fig 1:**
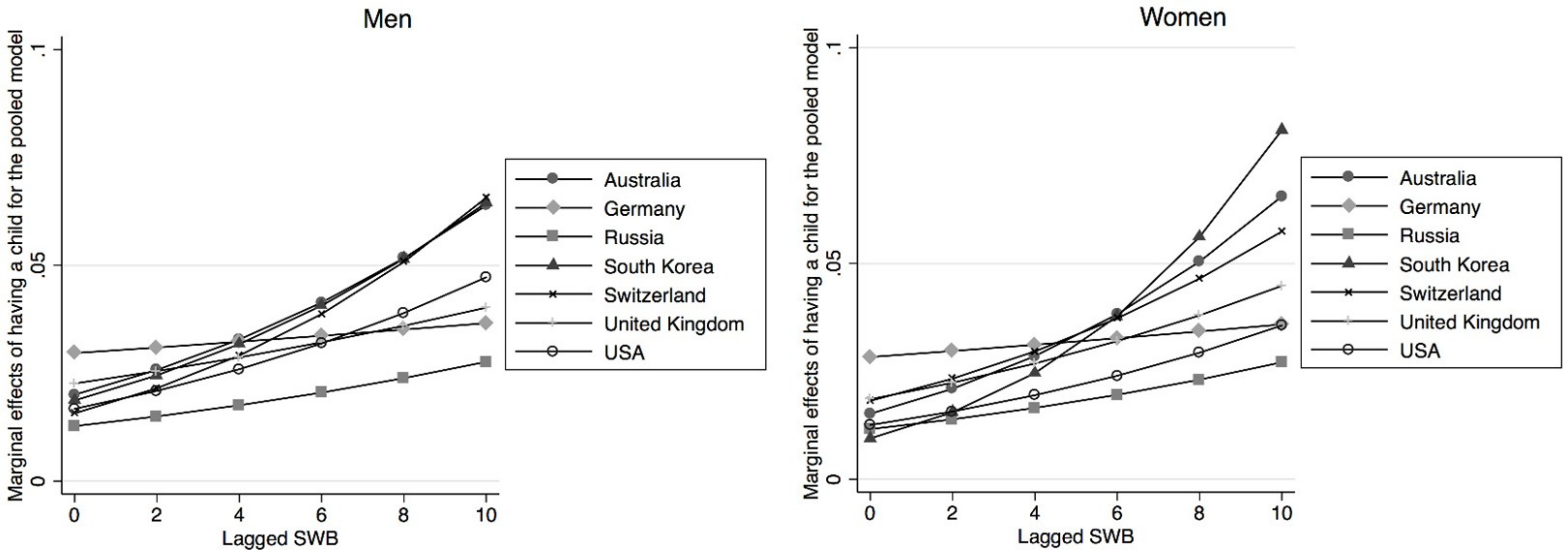
Marginal effects of having a child for the pooled model, by gender.

**Fig 2:**
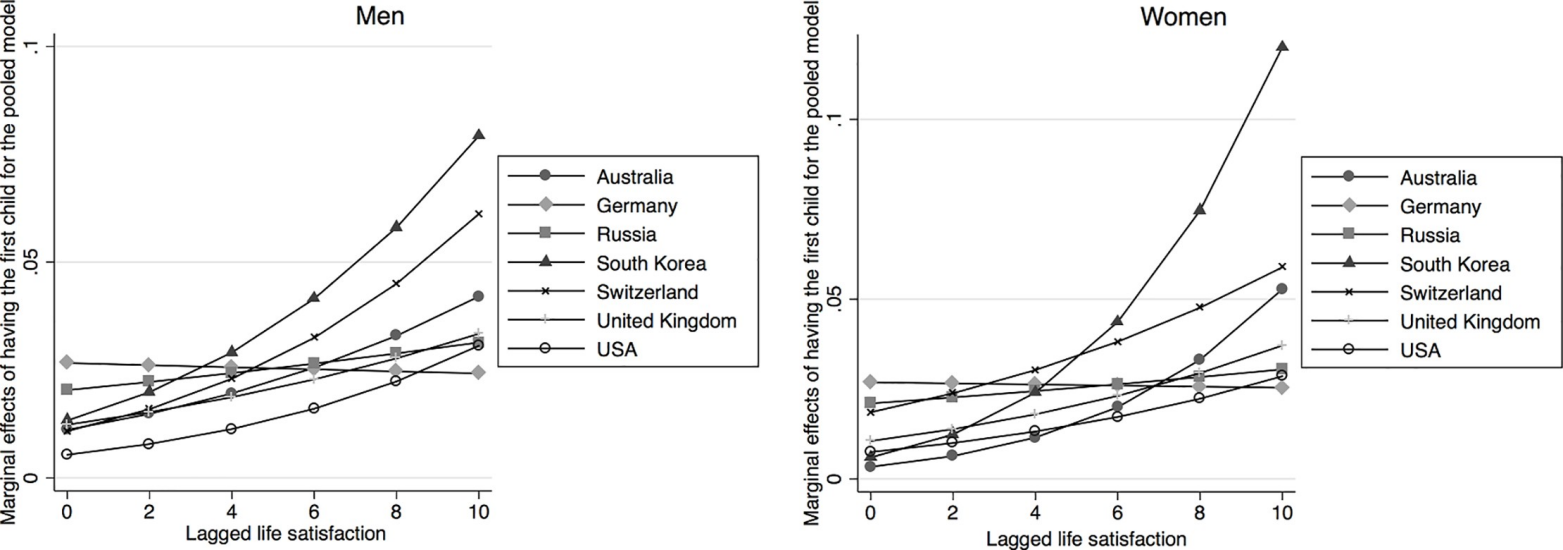
Marginal effects of having the first child for the pooled model, by gender.

**Fig 3:**
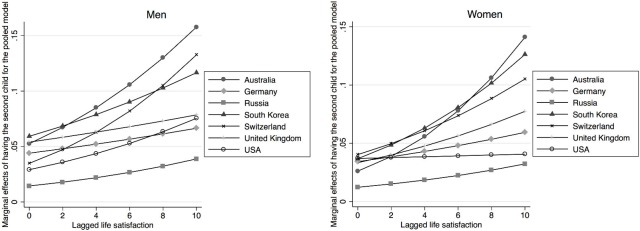
Marginal effects of having the second child for the pooled model, by gender.

Table 1 shows results from the pooled country regression. The estimation includes all explanatory variables described above, though they are not all reported in the table. The effects of the lagged value of life satisfaction is positive and highly significant for having any birth, the probability of having the first birth as well as for the second birth, and the effect is always stronger for women. As indicated above, the country dummy variables, together with interactions with those country dummies, test the extent to which there are country differences in the relationship between life satisfaction and the probability of childbearing. Since this is a non-linear model, any interpretation has to consider life satisfaction, the country dummies and the interaction between the two. Starting with the results for any birth, we see that the coefficients for Germany, Russia and for the United Kingdom are significantly different from that of Australia, which is the reference country. For the probability of having the first child, we also see a significant effect of life satisfaction for women in the Swiss sample, whereas for the probability of giving the second birth, we find a significant effect for US women, though there are now no difference between the Swiss sample and the reference country Australia. This means that there are some differences between countries in terms of the extent to which life satisfaction affects the probability of childbearing. However, the effects are mostly significantly positive and never negative. In order to better show the extent of these effects, and consequently highlight the differences across countries, we compute the effect of a step wise change in life satisfaction on the probability of having a birth, keeping the other explanatory variables fixed. These are commonly known as marginal effects and they are shown in in Fig 1 (the corresponding tables are available upon request). With computations done for the whole range of possible values of life satisfaction, Fig 1 indicates that the marginal effects are always positive. Consistent with the estimated coefficients, for low values of life satisfaction, the effect for Germany is the highest and the effect for Russia is the lowest. However, the marginal effects remain largely unaltered for these two countries as life satisfaction is increased. This is not the case with other countries, where the marginal effects increase with the level of life satisfaction. The strongest increase is found for Australia, South Korea, and Switzerland. The marginal effects for the first births are plotted in the next two graphs, in Fig 2. Here again we see that the marginal effects are positive for all countries: they are rather small for very low levels of life satisfaction, but they increase with higher levels of life satisfaction. The exceptions to this pattern are Germany and Russia, where the marginal effects remain similar across the values of life satisfaction. The final two graphs in Fig 3 show the marginal effects for the second birth. The trends are similar. The effects are positive, and they tend to be higher for larger values of life satisfaction. The trend of increasing effects is particularly strong for Australia, South Korea and Switzerland.

**Table 1:** Results of probit model on having a child (any birth order), first child and second child, by gender, pooled regression.

	Having a child		Having the first child		Having the second child	
	Men	Women	Men	Women	Men	Women
**Life Satisfaction (LS)**	0.0579***	0.0717***	0.0607***	0.119***	0.0677***	0.0965***
	(0.00993)	(0.00964)	(0.0154)	(0.0182)	(0.0186)	(0.0169)
**Interaction terms between life satisfaction and country**						
LS*Germany	-0.0476***	-0.0602***	-0.0651***	-0.121***	-0.0456**	-0.0674***
	(0.0113)	(0.0111)	(0.0176)	(0.0206)	(0.0205)	(0.0188)
LS*Russia	-0.0234*	-0.0342***	-0.0405**	-0.101***	-0.0222	-0.0525**
	(0.0127)	(0.0126)	(0.0194)	(0.0220)	(0.0223)	(0.0206)
LS*South Korea	-0.00781	0.0223	0.0121	0.00780	-0.0357	-0.0292
	(0.0170)	(0.0177)	(0.0256)	(0.0300)	(0.0321)	(0.0318)
LS*Switzerland	0.0123	-0.0153	0.0210	-0.0620*	0.00890	-0.0414
	(0.0227)	(0.0214)	(0.0308)	(0.0326)	(0.0437)	(0.0371)
LS*United Kingdom	-0.0302**	-0.0293**	-0.0157	-0.0620***	-0.0467*	-0.0509**
	(0.0128)	(0.0121)	(0.0197)	(0.0222)	(0.0239)	(0.0209)
LS*USA	-0.00841	-0.0242	0.0132	-0.0610	-0.0179	-0.0915**
	(0.0199)	(0.0232)	(0.0336)	(0.0463)	(0.0356)	(0.0358)
**Country fixed effects (Australia as reference category)**						
Germany	0.183**	0.283***	0.384***	0.849***	-0.0929	0.131
	(0.0897)	(0.0898)	(0.139)	(0.167)	(0.163)	(0.152)
Russia	-0.196**	-0.113	0.260*	0.735***	-0.612***	-0.337**
	(0.0922)	(0.0912)	(0.140)	(0.165)	(0.166)	(0.154)
South Korea	0.0248	-0.167	0.145	0.295	0.107	0.175
	(0.121)	(0.126)	(0.182)	(0.220)	(0.229)	(0.228)
Switzerland	-0.107	0.0790	-0.0115	0.680**	-0.211	0.214
	(0.185)	(0.177)	(0.251)	(0.268)	(0.357)	(0.309)
United Kingdom	0.0555	0.0880	0.0423	0.439**	0.0144	0.119
	(0.0977)	(0.0949)	(0.151)	(0.177)	(0.183)	(0.164)
USA	-0.0792	-0.0802	-0.289	0.301	-0.297	0.170
	(0.151)	(0.173)	(0.256)	(0.345)	(0.269)	(0.265)
Number of individuals	212,446	230,488	102,981	93,990	46,396	59,137

Note

*, **, *** indicate the 10%, 5%, and 1% significance levels, respectively.

Standard errors are clustered at the household level and reported in parentheses.

All the explanatory variables, which are stated in Tables 2, 3 and 4, are also controlled for.

**Fig 4:**
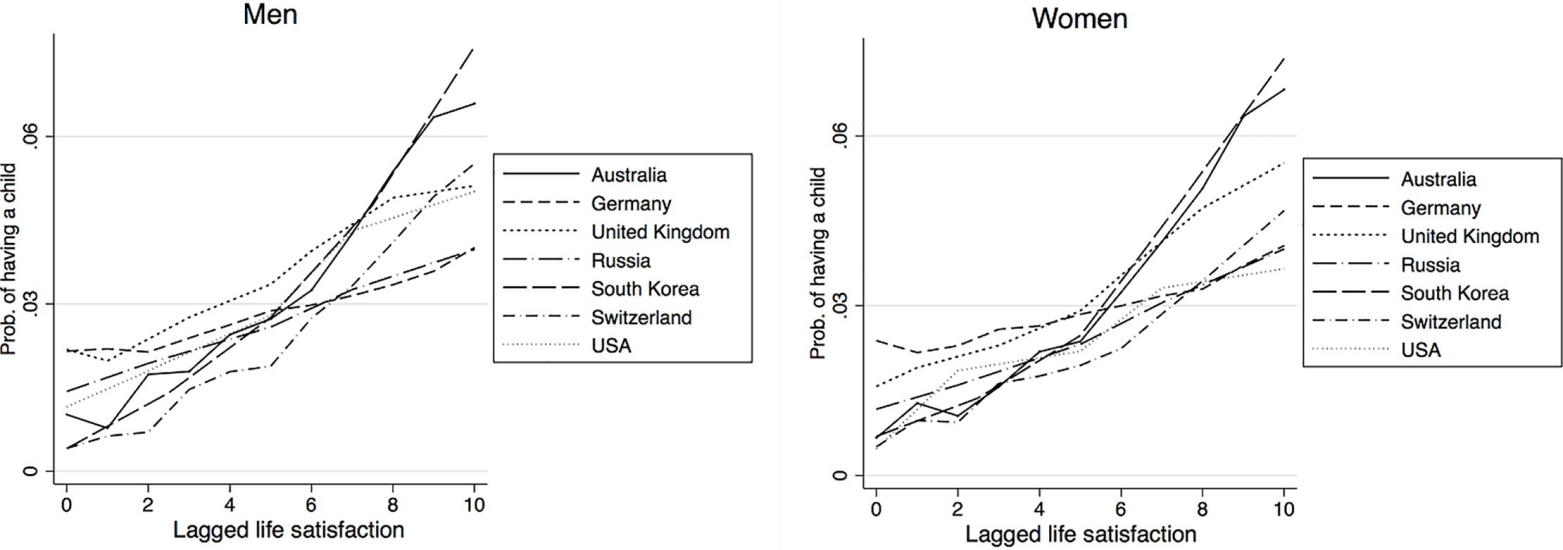
Predicted probabilities of having a child, by country and gender.

**Fig 5:**
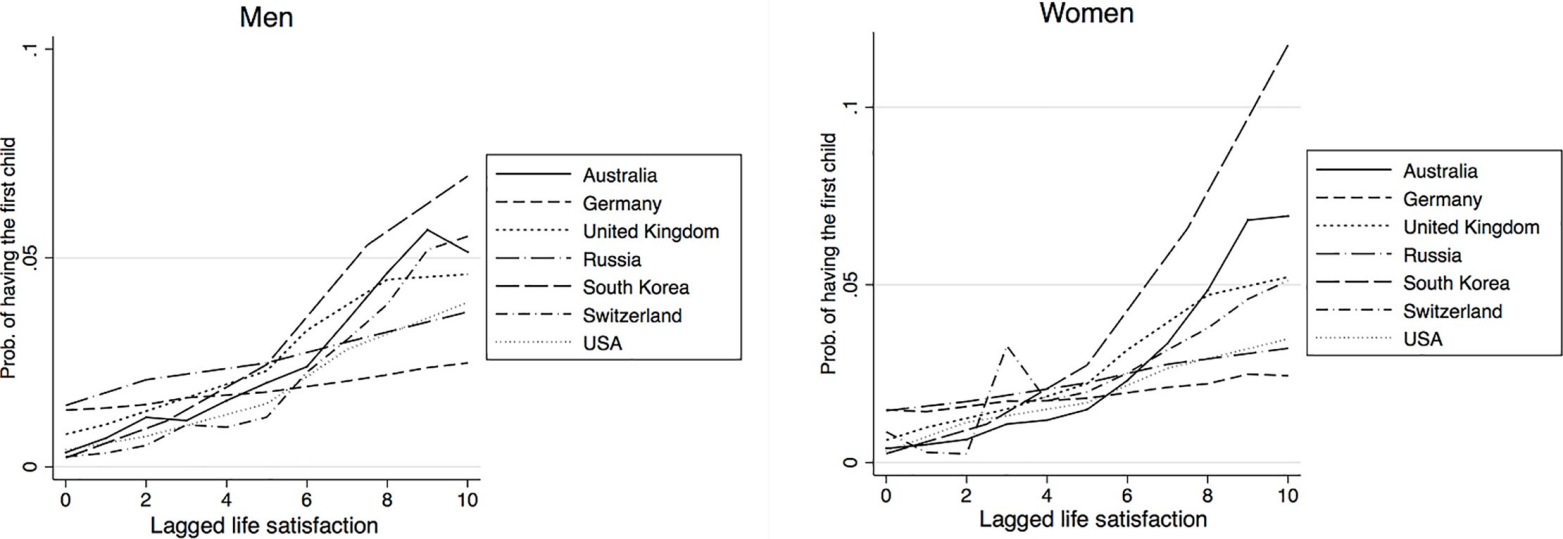
Predicted probabilities of having a first child, by country and gender.

**Fig 6:**
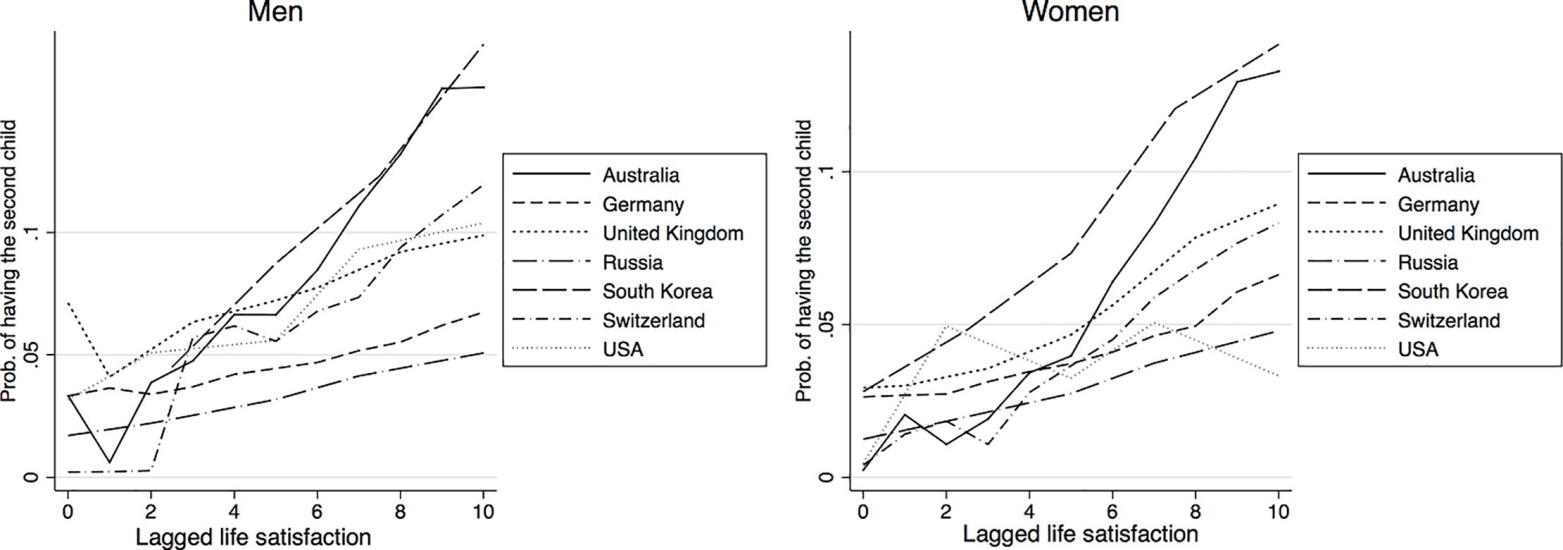
Predicted probabilities of having a second child, by country and gender.

Next we move on to the models where estimation is done separately for each country. The key difference between these models and the pooled regression reported previously, is that, in the latter, all coefficients of the background variables are restricted so as to be the same. In other words, the aim of the country-specific regressions, is to assess whether the effect of life satisfaction is still positive when there is no such restriction on the parameters. The results are shown in Tables 2, 3 and 4. As before, estimation is done separately by gender. The results are supplemented by Figs 4–6, which show the relationship between the level of life satisfaction (the lagged value being on the x-axis) and the predicted probabilities of having a(nother) child (on the y-axis). Again this is presented by gender. Across all countries we find that the higher the levels of subjective well-being, the higher the likelihood that couples will have children; this is true for both genders. The relationship is statistically significant and consistent for all countries and holds when we allow for country-specific coefficients on the control variables. Thus, an increase in the level of subjective well-being leads to an increase in the probability of having the first and the second child across countries for both genders. The positive effects on the likelihood of having a second child are generally more pronounced than the effects on the first child, especially in Germany and Russia. Taking these estimates, together with the pooled regression reported in Table 1, we might reasonably say that higher levels of subjective well-being are, indeed, associated with a higher probability of having children in all low fertility societies under consideration: again, this is true for both women and men. All these findings are valid net of the demographic and socio-economic characteristics of the couple.

**Table 2:** Results of probit model on having a child (of any birth order), by country and gender.

Table 2-a: Having a child														
	Australia		Germany		Russia		South Korea		Switzerland		United Kingdom		USA	
	Men	Women	Men	Women	Men	Women	Men	Women	Men	Women	Men	Women	Men	Women
Adj. Life Satisfaction	0.0538***	0.0712***	0.0172***	0.0225***	0.0240***	0.0280***	0.0277	0.0606***	0.0681***	0.0605***	0.0225***	0.0338***	0.0416**	0.0294
	(0.0106)	(0.0103)	(0.00536)	(0.00548)	(0.00785)	(0.00820)	(0.016)	(0.0168)	(0.0212)	(0.0212)	(0.00864)	(0.00791)	(0.0194)	(0.0238)
**Age Category**														
Age Group (20–29) (ref.category)														
Age Group (30–39)	-0.113***	-0.287***	-0.0994***	-0.313***	-0.485***	-0.705***	0.0789	-0.1183	0.165**	-0.200***	-0.313***	-0.422***	-0.233**	-0.285**
	(0.0310)	(0.0307)	(0.0246)	(0.0219)	(0.0506)	(0.0564)	(0.0697)	(0.062)	(0.0797)	(0.0683)	(0.0359)	(0.0338)	(0.114)	(0.131)
Age Group (40–49)	-0.988***	-1.715***	-0.703***	-0.788***	-0.753***	-0.728***	-0.6174***	-1.424***	-0.713***	-1.555***	-1.184***	-1.650***	-1.347***	-0.794***
	(0.0480)	(0.0835)	(0.0314)	(0.0291)	(0.0629)	(0.0578)	(0.0952)	(0.1300)	(0.103)	(0.118)	(0.0601)	(0.0897)	(0.268)	(0.182)
**In union**	0.835***	0.561***	0.495***	0.374***	0.392***	0.360***	1.063***	1.0946***	0.580***	0.632***	0.928***	0.603***	1.372***	1.419***
	(0.0470)	(0.0432)	(0.0334)	(0.0353)	(0.0783)	(0.0869)	(0.0835)	(0.1644)	(0.100)	(0.0933)	(0.0569)	(0.0453)	(0.177)	(0.206)
**Education Status**														
Primary Education	-0.014	-0.0581	0.042	0.0197	0.0708	0.0302	-0.1433	-0.6731	-0.264*	-0.033	-0.0338	0.0741	-0.290**	-0.262**
	(0.0370)	(0.0355)	(0.0281)	(0.0253)	(0.0540)	(0.0708)	(0.3518)	(0.5084)	(0.139)	(0.0954)	(0.0514)	(0.0509)	(0.122)	(0.131)
Secondary Education (ref.category)														
Tertiary Education	-0.00107	0.0629**	0.197***	0.165***	0.0365	0.0615	0.0729	0.0123	0.118**	0.245***	-0.00484	0.0758**	-0.0603	0.146
	(0.0328)	(0.0306)	(0.0252)	(0.0248)	(0.0482)	(0.0444)	(0.0506)	(0.0543)	(0.0590)	(0.0562)	(0.0349)	(0.0332)	(0.140)	(0.129)
**Employment Status**														
Employed	-0.0328	-0.247***	0.157***	-0.0907***	0.144**	0.0403	0.5742***	0.1363***	0.195*	0.104*	-0.0841	-0.183***	0.0239	-0.134
	(0.0532)	(0.0385)	(0.0317)	(0.0188)	(0.0605)	(0.0498)	(0.0878)	(0.0424)	(0.116)	(0.0624)	(0.0582)	(0.0346)	(0.117)	(0.103)
**Number of Children**														
No Child (ref.category)														
One Child	0.455***	0.471***	0.221***	0.231***	-0.141***	-0.171***	-0.3888***	-0.361***	0.221***	0.235***	0.275***	0.238***	0.359***	0.346***
	(0.0352)	(0.0353)	(0.0234)	(0.0222)	(0.0538)	(0.0518)	(0.0311)	(0.0323)	(0.0671)	(0.0661)	(0.0383)	(0.0373)	(0.114)	(0.122)
Two and more children	-0.230***	-0.135***	-0.0860***	-0.00655	-0.445***	-0.447***	-0.0545***	-0.0727***	-0.535***	-0.457***	-0.301***	-0.264***	0.204*	0.14
	(0.0393)	(0.0394)	(0.0281)	(0.0269)	(0.0757)	(0.0759)	(0.0088)	(0.0100)	(0.0805)	(0.0827)	(0.0437)	(0.0431)	(0.121)	(0.124)
**Household Income**														
1st Quintile	-0.157***	-0.0649	0.415***	0.411***	-0.274***	-0.186***	0.0306	0.0171	-0.124	-0.0742	-0.137**	-0.126**	-0.083	-0.0484
	(0.0544)	(0.0429)	(0.0298)	(0.0296)	(0.0670)	(0.0642)	(0.0695)	(0.0699)	(0.0934)	(0.0794)	(0.0588)	(0.0504)	(0.155)	(0.158)
2nd Quintile	-0.0252	-0.00842	0.116***	0.165***	-0.183**	-0.132*	-0.0776	-0.0737	-0.201**	-0.210***	-0.109**	-0.104**	-0.00892	-0.0439
	(0.0406)	(0.0450)	(0.0256)	(0.0256)	(0.0737)	(0.0735)	(0.0718)	(0.0715)	(0.0850)	(0.0802)	(0.0506)	(0.0482)	(0.150)	(0.150)
3rd Quintile (ref.category)														
4th Quintile	0.0317	0.0576	-0.122***	-0.0672**	0.024	0.0451	-0.0148	-0.0114	-0.157**	-0.106	-0.101**	-0.108**	0.0436	0.0652
	(0.0371)	(0.0496)	(0.0296)	(0.0279)	(0.0756)	(0.0752)	(0.0706)	(0.0723)	(0.0759)	(0.0761)	(0.0473)	(0.0476)	(0.131)	(0.136)
5th Quintile	-0.00534	-0.0375	-0.117***	-0.0757**	-0.0123	-0.0573	0.0192	-0.1524**	-0.0256	0.0853	0.00415	0.0412	0.207	0.0565
	(0.0441)	(0.0564)	(0.0329)	(0.0323)	(0.0606)	(0.0612)	(0.0746)	(0.0752)	(0.0741)	(0.0734)	(0.0481)	(0.0455)	(0.136)	(0.145)
**Partner’s Education Status**														
Primary Education	-0.193***	-0.141***	0.021	-0.00138	0.0859	0.0694	-0.2236	0.1859	-0.0863	-0.125	-0.162*	-0.187**	0.0397	-0.0467
	(0.0414)	(0.0432)	(0.0279)	(0.0305)	(0.0799)	(0.0633)	(0.4524)	(0.3722)	(0.104)	(0.146)	(0.0867)	(0.0758)	(0.144)	(0.141)
Secondary Education (ref.category)														
Tertiary Education	0.135***	0.0865**	0.106***	0.122***	0.176***	0.0734	0.1154**	0.0938	0.0659	0.0167	0.162***	-0.0314	0.0837	0.122
	(0.0340)	(0.0351)	(0.0293)	(0.0284)	(0.0523)	(0.0556)	(0.0586)	(0.0598)	(0.0726)	(0.0657)	(0.0465)	(0.0509)	(0.128)	(0.117)
**Partner’s Employment Status**														
Employed	-0.0972***	0.0249	-0.0727***	-0.0285	-0.0335	0.0405	-0.0279	0.1302	-0.0471	-0.036	-0.125***	0.0191	-0.258***	-0.157
	(0.0303)	(0.0346)	(0.0228)	(0.0295)	(0.0569)	(0.0715)	(0.0517)	(0.1486)	(0.0658)	(0.0693)	(0.0428)	(0.0448)	(0.0981)	(0.118)
Number of individuals	33049	35721	105793	111372	18482	21379	15940	16031	9789	12572	25623	29519	3766	3890

Notes

*, **, *** indicate the 10%, 5%, and 1% significance levels, respectively. Standard errors are clustered at the household level and reported in parentheses. Results are controlled for region and year survey dummies

**Table 3:** Results of probit model on having the first child, by country and gender.

Table 2-b: Having the first child														
	Australia		Germany		Russia		South Korea		Switzerland		United Kingdom		USA	
	Men	Women	Men	Women	Men	Women	Men	Women	Men	Women	Men	Women	Men	Women
**Adj. Life Satisfaction**	0.0596***	0.122***	0.0085	0.00874	0.00658	0.00879	0.0273	0.088***	0.0828***	0.0584*	0.0395***	0.0478***	0.0850***	0.0553
	(0.0162)	(0.0191)	(0.00833)	(0.00937)	(0.0120)	(0.0127)	(0.0234)	(0.0264)**	(0.0287)	(0.0305)	(0.0132)	(0.0140)	(0.0318)	(0.0417)
**Age Category**														
Age Group (20–29) (ref.category)														
Age Group (30–39)	0.0466	0.0826*	0.121***	-0.127***	-0.798***	-0.929***	0.1474	0.0843	0.273***	0.0692	-0.181***	-0.243***	-0.0709	-0.211
	(0.0409)	(0.0465)	(0.0344)	(0.0411)	(0.104)	(0.104)	(0.0799)	(0.0767)	(0.0848)	(0.0803)	(0.0513)	(0.0565)	(0.165)	(0.183)
Age Group (40–49)	-0.800***	-1.561***	-0.535***	-0.644***	-0.890***	-0.875***	-0.2594	-1.4713***	-0.651***	-1.523***	-1.275***	-1.877***	-1.199***	-0.940***
	(0.0753)	(0.151)	(0.0478)	(0.0442)	(0.0856)	(0.0768)	(0.1418)	(0.3556)	(0.139)	(0.213)	(0.106)	(0.176)	(0.375)	(0.281)
**In union**	0.935***	0.937***	0.436***	0.466***	0.551***	0.589***	0.7185***	0.7475***	0.656***	0.804***	0.876***	0.839***	1.042***	1.306***
	(0.0603)	(0.0704)	(0.0545)	(0.0574)	(0.125)	(0.138)	(0.1290)	(0.2621)	(0.142)	(0.133)	(0.0658)	(0.0703)	(0.264)	(0.291)
**Education Status**														
Primary Education	-0.00267	-0.0317	0.0294	0.00044	-0.111	0.174	-0.2454		-0.282*	-0.00289	-0.0224	0.0363	-0.0517	-0.539
	(0.0536)	(0.0731)	(0.0440)	(0.0455)	(0.0903)	(0.108)	(0.434)		(0.169)	(0.137)	(0.0820)	(0.108)	(0.238)	(0.415)
Secondary Education (ref.category)														
Tertiary Education	-0.0192	0.0914*	0.140***	0.125***	-0.0429	0.0739	0.1119	-0.1253	0.0805	0.223***	-0.0679	0.0006	-0.076	0.0763
	(0.0458)	(0.0470)	(0.0363)	(0.0388)	(0.0701)	(0.0704)	(0.0761)	-0.0854	(0.0787)	(0.0766)	(0.0517)	(0.0536)	(0.190)	(0.160)
**Employment Status**														
Employed	0.0482	-0.311***	0.232***	0.110***	0.302***	0.140*	0.5681***	0.3257***	0.248	0.319**	0.108	0.179**	0.122	-0.196
	(0.0753)	(0.0715)	(0.0457)	(0.0376)	(0.0909)	(0.0838)	(0.1038)	(0.0709)	(0.156)	(0.140)	(0.0943)	(0.0884)	(0.181)	(0.173)
**Household Income**														
1st Quintile	-0.219***	-0.179**	0.335***	0.334***	-0.105	-0.0144	0.1359	0.0633	-0.389	-0.384*	0.0684	0.144	0.0518	-0.227
	(0.0783)	(0.0768)	(0.0455)	(0.0488)	(0.0978)	(0.103)	(0.1262)	(0.1207)	(0.255)	(0.222)	(0.0945)	(0.0963)	(0.244)	(0.326)
2nd Quintile	-0.0462	0.0226	0.0607	0.117**	-0.0772	-0.0279	0.1611	0.0859	-0.2	-0.552**	-0.00276	0.0283	0.254	0.0555
	(0.0600)	(0.0643)	(0.0452)	(0.0467)	(0.109)	(0.121)	(0.1217)	(0.1191)	(0.175)	(0.238)	(0.0808)	(0.0862)	(0.231)	(0.261)
3rd Quintile (ref.category)														
4th Quintile	0.0192	0.00508	-0.0965**	-0.0168	0.0623	0.0231	0.2985**	0.2718**	-0.0934	-0.144	-0.0376	-0.0744	-0.712*	-0.0719
	(0.0545)	(0.0704)	(0.0445)	(0.0443)	(0.114)	(0.119)	(0.1160)	(0.1131)	(0.0975)	(0.104)	(0.0711)	(0.0786)	(0.394)	(0.225)
5th Quintile	-0.0765	-0.0613	-0.0959**	-0.0234	0.208**	0.0714	0.2823**	-0.047	0.0684	0.0673	0.0851	0.125*	0.356*	0.06
	(0.0622)	(0.0748)	(0.0448)	(0.0459)	(0.0950)	(0.102)	(0.1213)	(0.1191)	(0.0867)	(0.0892)	(0.0698)	(0.0684)	(0.195)	(0.227)
**Partner’s Education Status**														
Primary Education	-0.0916	-0.161**	0.0239	-0.062	0.19	-0.178		1.4412**	-0.113	0.014	-0.16	-0.237	-0.0288	-0.203
	(0.0694)	(0.0761)	(0.0563)	(0.0612)	(0.134)	(0.113)		(0.6962)	(0.203)	(0.236)	(0.158)	(0.154)	(0.262)	(0.258)
Secondary Education (ref.category)														
Tertiary Education	0.156***	0.000464	0.0955*	0.0182	0.237***	-0.00539	0.2006	0.2505	0.0543	-0.0998	0.0783	-0.108	0.0301	0.0509
	(0.0506)	(0.0550)	(0.0549)	(0.0504)	(0.0858)	(0.0955)	(0.129)	(0.1348)	(0.127)	(0.133)	(0.0733)	(0.0832)	(0.227)	(0.165)
**Partner’s Employment Status**														
Employed	-0.0812	0.0605	0.0297	-0.0427	-0.0267	0.0968	0.0924	0.0798	0.04	-0.105	0.0359	0.0614	-0.0962	-0.17
	(0.0500)	(0.0572)	(0.0504)	(0.0520)	(0.107)	(0.117)	(0.1117)	(0.2481)	(0.139)	(0.137)	(0.0668)	(0.0731)	(0.189)	(0.185)
Number of individuals	17,484	13,730	50,825	47,429	8,248	8,725	6,944	5,231	4,526	4,764	13,110	12,398	1,829	1,660

Notes

*, **, *** indicate the 10%, 5%, and 1% significance levels, respectively. Standard errors are clustered at the household level and reported in parentheses. Results are controlled for region and year survey dum

**Table 4:** Results of probit model on having the second child, by country and gender.

Table 2-b: Having the second child														
	Australia		Germany		Russia		South Korea		Switzerland		United Kingdom		USA	
	Men	Women	Men	Women	Men	Women	Men	Women	Men	Women	Men	Women	Men	Women
**Adj. Life Satisfaction**	0.0606***	0.0859***	0.0159*	0.0249***	0.0342***	0.0316***	0.0264	0.0559*	0.0414	0.0376	-0.00577	0.00259	0.0627*	0.00608
	(0.0198)	(0.0187)	(0.00885)	(0.00826)	(0.0124)	(0.0122)	(0.0286)	(0.0298)	(0.0416)	(0.0375)	(0.0176)	(0.0147)	(0.0376)	(0.0387)
**Age Category**														
Age Group (20–29) (ref.category)														
Age Group (30–39)	-0.211***	-0.295***	-0.0272	-0.208***	-0.260***	-0.582***	-0.2401	-0.2861***	0.285**	-0.0142	-0.149**	-0.271***	-0.504*	-0.252
	(0.0574)	(0.0533)	(0.0378)	(0.0343)	(0.0826)	(0.0907)	(-0.1329)	(0.0939)	(0.132)	(0.108)	(0.0722)	(0.0658)	(0.278)	(0.238)
Age Group (40–49)	-1.130***	-2.153***	-0.787***	-0.826***	-0.796***	-0.728***	-1.0793***	-1.6317***	-0.571***	-1.381***	-0.560***	-1.269***	0.587***	-0.604**
	(0.0837)	(0.186)	(0.0552)	(0.0552)	(0.138)	(0.124)	(0.1610)	(0.2026)	(0.175)	(0.176)	(0.121)	(0.210)	(0.180)	(0.283)
**In union**	0.493***	0.455***	0.485***	0.293***	0.249**	0.313**	0.4178**	0.4376	0.467***	0.605***	0.435**	0.400***	1.688***	1.562***
	(0.103)	(0.0797)	(0.0534)	(0.0521)	(0.117)	(0.150)	(0.2062)	(0.3545)	(0.175)	(0.149)	(0.190)	(0.0888)	(0.350)	(0.406)
**Education Status**														
Primary Education	-0.183**	-0.176***	-0.0118	-0.0453	0.182**	-0.111			-0.360	-0.0268	-0.0873	0.0781	-0.267	-0.447**
	(0.0721)	(0.0648)	(0.0430)	(0.0386)	(0.0787)	(0.104)			(0.246)	(0.165)	(0.109)	(0.105)	(0.191)	(0.197)
Secondary Education (ref.category)														
Tertiary Education	0.0155	-0.0228	0.249***	0.177***	0.0704	0.0847	0.0898	(0.0852)	0.189*	0.217**	0.0857	0.0957	0.483*	0.187
	(0.0686)	(0.0626)	(0.0405)	(0.0373)	(0.0743)	(0.0639)	(0.0878)	(0.0908)	(0.105)	(0.0963)	(0.0711)	(0.0641)	(0.274)	(0.218)
**Employment Status**														
Employed	-0.00504	-0.0163	0.174***	-0.0360	-0.00257	0.0477	0.1661	0.0639	0.205	0.190*	0.0772	-0.0526	0.00824	0.266
	(0.108)	(0.0740)	(0.0530)	(0.0286)	(0.0922)	(0.0776)	(0.2087)	(0.0851)	(0.232)	(0.105)	(0.120)	(0.0644)	(0.229)	(0.186)
**Age of the youngest child**	-0.0440***	-0.0468***	-0.0594***	-0.0597***	-0.0372***	-0.0277**	-0.0929***	-0.1053***	-0.123***	-0.0963***	-0.114***	-0.103***	-0.0950**	-0.0838**
	(0.00877)	(0.00765)	(0.00433)	(0.00378)	(0.0119)	(0.0122)	(0.0148)	(0.0162)	(0.0232)	(0.0217)	(0.00928)	(0.00846)	(0.0428)	(0.0333)
**Household Income**														
1st Quintile	-0.0827	0.0254	0.329***	0.350***	-0.327***	-0.264***	-0.0965	-0.1446	-0.106	-0.163	0.104	-0.0489	0.127	0.429
	(0.107)	(0.0821)	(0.0457)	(0.0422)	(0.108)	(0.0985)	(0.1223)	(0.125)	(0.190)	(0.162)	(0.113)	(0.100)	(0.315)	(0.289)
2nd Quintile	-0.0327	-0.029	0.0858**	0.155***	-0.211*	-0.151	-0.1781	-0.1732	-0.155	-0.199	-0.0441	-0.0364	-0.262	0.253
	(0.0787)	(0.0930)	(0.0434)	(0.0410)	(0.117)	(0.105)	(0.1307)	(0.1276)	(0.152)	(0.136)	(0.101)	(0.0974)	(0.324)	(0.241)
3rd Quintile (ref.category)														
4th Quintile	-0.0327	-0.029	-0.118**	-0.104**	0.0726	0.114	-0.2035	-0.1406	-0.129	-0.123	-0.0878	0.0725	0.246	0.334
	(0.0787)	(0.0930)	(0.0479)	(0.0460)	(0.113)	(0.106)	(0.1336)	(0.1346)	(0.138)	(0.135)	(0.0910)	(0.0882)	(0.226)	(0.237)
5th Quintile	0.0281	-0.133	-0.127**	-0.121**	-0.0926	-0.0551	-0.0973	-0.0756	-0.317	-0.0759	-0.113	0.0239	0.0381	-0.0676
	(0.0907)	(0.129)	(0.0571)	(0.0553)	(0.0921)	(0.0891)	(0.1383)	(0.1354)	(0.194)	(0.160)	(0.0981)	(0.0913)	(0.247)	(0.262)
**Partner’s Education Status**														
Primary Education	-0.334***	-0.239***	-0.0109	-0.0119	0.0303	0.165*			-0.0731	-0.161	-0.355**	-0.0241	0.357	0.165
	(0.0725)	(0.0810)	(0.0410)	(0.0435)	(0.113)	(0.0908)			(0.201)	(0.274)	(0.173)	(0.153)	(0.275)	(0.240)
Secondary Education (ref.category)														
Tertiary Education	0.0701	0.0899	0.105**	0.149***	0.129*	0.0513	0.0715	0.05	-0.0769	0.0276	0.112	0.151	-0.0836	0.0266
	(0.0677)	(0.0687)	(0.0434)	(0.0422)	(0.0750)	(0.0796)	(0.0881)	(0.0912)	(0.109)	(0.103)	(0.0836)	(0.0985)	(0.214)	(0.207)
Partner’s Employment Status														
**Employed**	0.0148	0.0513	-0.0671**	0.0715*	0.0898	0.0556	0.1053	0.2291	0.0177	-0.0681	-0.117	-0.00455	0.0677	0.0451
	(0.0554)	(0.0644)	(0.0334)	(0.0433)	(0.0813)	(0.114)	(0.0854)	(0.2153)	(0.112)	(0.112)	(0.0798)	(0.0889)	(0.192)	(0.215)
Number of individuals	4,781	6,752	25,144	30,811	6,828	8,626	2,338	2,511	1,527	2,315	4,337	6,307	622	994

Notes

*, **, *** indicate the 10%, 5%, and 1% significance levels, respectively. Standard errors are clustered at the household level and reported in parentheses. Results are controlled for region and year survey dummies.

As with the pooled regression, one can also see here some differences between countries. Whereas the overall effect of life satisfaction on fertility is positive for all countries, we find that for Australia and the United Kingdom, increasing levels of subjective well-being positively predict the probability of having a child of any order, and the first child for both women and men; while, for the second child, the results lose their precision. In Germany, the coefficient of subjective well-being for both having a child and having the second child are positive and significant. In Switzerland and the USA, the effects of life satisfaction on fertility are more pronounced for men than for women. For the South Korean sample, we find a positive effect of life satisfaction on the probability of having a child. When looking at the probability of a second child, the effect is still positive, but there is no longer statistical precision. The estimates of life satisfaction for Russia are flatter than those for other countries for having the first and the second child. Despite these differences, the countries are similar in that life satisfaction positively predicts higher fertility.

When we analyze the average marginal effect by country for women, we observe the highest score for Australia: moving from six to nine on the life satisfaction scale gives a marginal effect of 0.06 in the probability of having a child. The statistical model estimates the determinants of the probability of giving birth in one year. To better grasp the effects size, it is useful to contrast a one-year temporary rise as opposed to a permanent rise in life satisfaction. For example, the marginal effect of 0.06 found for Australia means that a temporary increase in life satisfaction from six to nine for one year would lead to an increase in number of births by 0.06 in that year. In contrast, a permanent increase in life satisfaction taking place at age 20 and lasting for 30 years (which would refer to the complete reproductive age spans from 20 to 50), would increase the number of births by 1.8 (= 30×0.06). This is a rather dramatic effect, but it is also unlikely that an increase in life satisfaction remains permanent for such a long period. For instance, an increase that remained for 10 years instead, would increase the number of births by 0.6. With this perspective in mind, we can move on to discuss the country differences.

The lowest score is for Russia and the US: the average marginal effect from six to nine scale is 0.005 in the probability of having a child. For men we have the similar pattern but the average marginal effect for Australia from six to nine is around 0.05 in the probability of having a child, which has a small drop compared to women. Switzerland is the only country where the average marginal effect from seven to ten is higher for women (0.045) than men (0.03) in the probability of having a child. For the UK, the marginal effect of probability of having the first child is 0.008, which is higher than probability of having a child (the effect is 0.003) for women from scale seven to eight. In the probability of having a second child, the largest marginal effect, which is 0.025, from six to nine scale records for German women.

The results for our demographic controls are in line with standard outcomes. Clearly, the older age groups negatively predict the probability of having a child, and the coefficients are significant for both men and women in all countries. As expected, the effect of living in a co-resident union is positive and statistically significant in all specifications. Having two or more children is significantly negatively associated with the probability of having another child in all countries, except the USA. On the other hand, having one child is strongly positively and significantly related with the probability of having a subsequent child in all countries but Russia. The effect of the age of the youngest child is the same across societies and for men and women: it is negative and statistically significant for having a second child.

As for the socio-economic predictors, the level of education produces mixed results by country, with a significant positive effect on predicting the probability of having a child among more highly-educated individuals in Germany and Switzerland. There is, though, a significant negative effect on those with just primary education in Australia and the USA (only for the second child). For South Korea, we find that women with higher education have a lower likelihood of having the first child–otherwise there is no significant effect of educational qualifications.

Employment status produces different results by gender. Overall, the link between labor-force participation and childbearing seems to be positive for men and negative for women. Women’s employment negatively affects fertility, especially as regards the probability of having a second child. Interestingly, women’s employment seems to be positively linked to the probability of having a first child in Germany, Russia, Switzerland, and the United Kingdom.

Not only respondents, but also partners’ socio-economic characteristics play a key role in directing fertility behavior. A partner’s educational level is crucial for having a child: having a partner with higher education increases the probability of having a child for both genders. The effect is remarkable in Australia, Germany and South Korea. Likewise, partners with only primary education predicts fertility negatively, and it is statistically significant for the United Kingdom and Australia and for South Korea for the probability of having the second birth. As for the effect of a partners’ employment status, results show that, for women, having an employed partner has a positive effect on fertility. For South Korea, the effect is particularly strong on the probability of having the second birth. For men, on the other hand, having a partner or wife who is employed is negatively and significantly related to having a child in Australia, Germany, South Korea, the United Kingdom, and the USA. Broadly speaking, as with the household income quintiles, belonging to lower-income households predicts positively and significantly the probability of having a child in Germany and negatively in Russia and the United Kingdom.

To verify whether our findings are replicated among different social groups, we stratified the analysis by educational qualifications (results not shown but available upon request). We found a virtually unchanged pattern among women with secondary and tertiary education. Interestingly, among women with primary education alone, the positive association between life satisfaction and the probability of having a child loses its significance. In Germany, there are even indications of a negative, marginally significant, association. Hence, besides the general positive impact of life satisfaction on fertility, the link seems to be more fragile among individuals belonging to more disadvantaged social groups. A possible explanation for this finding may be the “uncertainty reduction” narrative inspired by Friedman et al. [42]: individuals with an uncertain outlook may decide to have a child to increase their life satisfaction.

The robustness of the regression results, as well as the validation of the model, have been verified through a series of sensitivity analyses (see the Appendix 1). These confirm that our results are not sensitive to various model specifications and sample restrictions.

## Discussion

The extent to which subjective well-being affects fertility levels has so far received little scholarly attention and it has never previously been addressed in a comparative framework [43]. This paper investigates whether life satisfaction brings about a higher likelihood of childbearing. We posit that in developed countries, this positive relationship is universal. In contrast to developing countries, childbearing in developed countries is, under most circumstances, a conscious choice. Some single-country studies have hinted at this result already. The present study is the first of its kind in that it provides a systematic analysis of large and long-running longitudinal surveys across seven major low-fertility countries: Australia, Germany, Russia, South Korea, Switzerland, the United Kingdom, and the United States. The novelty of the study lies in providing new, robust comparative empirical evidence of the relationship between subjective well-being and fertility.

We found that, in the seven countries, relatively higher levels of life satisfaction do, indeed, foster reproductive behavior. The fact that higher levels of cognitive subjective well-being are associated with a higher probability of having children in all countries under consideration–with some differences in the degree of association among them–suggests that life satisfaction favors reproduction. Where childbearing has become optional, financially expensive, and is associated with considerable trade-offs in terms of professional careers and other life goals, childbearing is not “out of fashion”. Indeed, it remains an important life-experience for most adults. However, it tends to be attempted only in conjunction with a satisfying life.

Why do these results matter? Low fertility is one of the most pressing issues in industrialized nations. It is a key driver of our ageing societies, and hence it strains social-security systems. After the baby boom of the 1960s and the 1970s, fertility levels collapsed in most developed countries of the developed world. In Europe, the total fertility rate fell below the replacement rate in most countries, but it became extremely low in Southern and Eastern Europe, where the fertility rate touched 1.3 children per woman. Other countries followed suit, notably Japan and South Korea, the countries with, today, the lowest fertility levels in the world. Against this backdrop, economists assumed that women’s education and employment increase the opportunity cost of childrearing, which depressed fertility [44]. Sociological approaches refer to similar mechanisms as evidence of “role incompatibility”–i.e. the inability to combine mother and worker roles in a modern economy where home and workplace are separated [45]. The demographer van de Kaa [21] noted, meanwhile, that trends towards lowest-low fertility levels appeared in a period where individuals changed their attitudes towards childbearing: self-realization and fulfillment of one’s well-being took center stage giving rise to a new societal landscape. Having children was given lower priority than had been the case in the past. This line of thought is often referred to as the Second Demographic Transition [21–22]. However, available data suggests that something is missing from the picture. Today, the countries that have progressed furthest on the path of the Second Demographic Transition are also the countries with the highest fertility rates among rich societies. They are also the countries with the highest reported levels of subjective well-being [46, 47]. The thesis, then, that life satisfaction brings about higher fertility promises to fill an important gap in population theory. The present study goes a long way to suggesting that this is, indeed, the case, and that increased subjective well-being should be viewed as a policy objective that could increase fertility.

Previous studies have argued for a positive association between fertility rates and subjective well-being, though with some important differences by country and by parity. Essentially these studies argued that the better the institutional support for reconciling work and family life, the happier people are when having children, and hence the higher fertility rates. Even though societal features play a role in shaping the life satisfaction-fertility relationship, the cross-sectional nature of those studies (e.g. the European Social Survey in [47–48]) did not allow inferences about whether subjective well-being affects fertility or whether fertility affects subjective well-being. This study gives a definite answer here. It suggests that part of that positive relationship comes from the fact that a higher level of life satisfaction explains higher subsequent fertility.

Another important contribution of this paper is that we consider the effects by parity, i.e. how the potential effect of subjective well-being on childbearing differs according to whether we consider the first or the second birth. The positive effects of life satisfaction on the probability of having a second child are more pronounced than the ones for a first child, notably in Germany, Russia and South Korea, the lowest fertility countries of the seven under consideration (levels below 1.5 children per women). If the experience of having a first child is more difficult than parents had foreseen, subjective well-being may decrease. Hence, where fertility is worryingly low, this might be partly a result of the unsatisfactory (i.e. unhappy) experience of childrearing, especially when the reconciliation between work, family and young children proves difficult [49].

Does life satisfaction thus influence the decision to have children? An extensive comparative analysis of longitudinal data suggests that yes, it does.

## Appendix: Sensitivity analysis

It was crucial to test our findings with a series of sensitivity checks (they are not shown here, but are available upon request). First, we verified the stability of our results after restricting the sample to those individuals who are in a union. The results show that the effects of subjective well-being on fertility remains virtually unchanged after such sample restrictions were introduced. Second, to avoid the anticipation effect, i.e. the change in the level of life satisfaction due to pregnancy status, we performed a sensitivity check in which we re-estimated the model by lagging values back to t-3. The results under all specifications yield very similar outcomes to lagged values at t-2. Third, our results are not sensitive to different age specifications–i.e. considering different cut-off points (such as a five-year age group specification) or a continuous variant of age. Finally, we need to stress that our econometric framework comprises a traditional probit model, which does not take into account unobservable time-invariant factors. Our estimates could, thus, be objected to. Nonetheless, fixed effects cannot typically be added to a probit model without introducing bias into the coefficients and standard errors [50]. There are two possible ways to extend our analyses using a fixed effects estimation strategy in this context. One would be to use a linear probability model to estimate the marginal effect, instead of a probit model because linear models are not typically subject to incidental parameters bias [50–51]. In such cases, however, one might encounter several other problems in dealing with binary outcome variables such as heteroskedastic standard errors and nonsensical predictions (i.e. predicted probabilities outside the zero to one interval). The second way is to employ a logit instead of a probit model, and to utilize Chamberlain’s method of conditional fixed effects. This method still carries a bias in the coefficients. Research on resampling methods has also shown that a jackknife estimator can be used to reduce bias in fixed effects probit models [52]. We re-estimated our models by following all the above mentioned strategies. Results suggest that incising levels of subjective well-being are positively linked with the likelihood of childbearing in a very similar fashion to the results without fixed effects.
